# Characteristics of adult abdominal cystic Lymphangioma: a single-center Chinese cohort of 12 cases

**DOI:** 10.1186/s12876-020-01388-8

**Published:** 2020-07-29

**Authors:** Jianchun Xiao, Yuming Shao, Shan Zhu, Xiaodong He

**Affiliations:** 1 Department of General Surgery, Peking Union Medical College Hospital, Chinese Academy of Medical Sciences & Peking Union Medical College, No. 1 Shuaifuyuan, Beijing, 100730 China; 2 Institute of Clinical Medicine, Peking Union Medical College Hospital, Chinese Academy of Medical Sciences & Peking Union Medical College, Beijing, China; 3 Department of Obstetrics and Gynecology, Peking Union Medical College Hospital, Chinese Academy of Medical Sciences & Peking Union Medical College, Beijing, China

**Keywords:** Abdominal cystic lymphangioma, Adult, Cystic lymphangioma, Surgery

## Abstract

**Background:**

Cystic lymphangioma is a rare, benign developmental disease, mostly affecting the cervical and axial regions. The clinical features of abdominal cystic lymphangioma (ACL) are reported among pediatric patients but are less well known in adults. The purpose of this study was to demonstrate the clinical characteristics of ACL in Chinese adults and describe our experience in treating this disease.

**Methods:**

We conducted a single-center, non-interventional, retrospective study of 12 adult patients with ACL admitted to Peking Union Medical College Hospital in Beijing, China from November 1984 through August 2017. The demographic, clinical, laboratory, imaging, histopathologic, and therapeutic data were collected.

**Results:**

Detailed information on seven males and five females was available. The mean age at diagnosis was 39.1 (SD 17.3) years. The mean duration of follow-up was 6.9 years. Six (50%) patients were asymptomatic, and abdominal pain was the leading symptom for three (25%) patients. The cysts were evaluated by ultrasound (*n* = 8, 67%), CT (*n* = 10, 83%), and MRI (*n* = 4, 33%). Therapeutic modalities include laparotomy (*n* = 6, 50%), laparoscopy (n = 4, 33%), and aspiration (*n* = 2, 17%), with variable outcomes. The mean postsurgical hospital stay was 6.8 days. Complete excision was accomplished in eight patients, and one recurrence was observed during follow-up. Of patients who underwent partial resection, one experienced disease relapse.

**Conclusions:**

This is the first report on an Asian cohort of adult patients with ACL. Typical imaging features could lead to timely diagnosis and treatment of ACL. Radical resection is recommended with a longer period of follow-up. The analysis of this cohort deepens our understanding of adult ACL.

## Background

Cystic lymphangioma (CL) is an underestimated, benign developmental disease. The suggested etiology of CL is an incorrect embryological connection of the lymphatics when primary lymphatic cysts fail to converge with the main lymphatic system [1, 2]. CL may develop in a variety of anatomic locations; while a majority of CL locate in the cervical and axial regions [3]. Abdominal cystic lymphangioma (ACL) is an even rarer entity, accounting for less than 5% of all CL cases [4]. There are several reports on the clinical characteristics of ACL, mostly on pediatric patients [5, 6].

The clinical features of ACL in adults remain unclear; the real prevalence in adults may be vastly underestimated because of a lack of recognition of the disease or an atypical clinical presentations. It may be misdiagnosed as a pancreatic pseudocyst, ovarian cyst, renal cyst, or other disease based on its location. Currently, most articles on ACL are case reports or description of small cohorts [7, 8]. We aimed to describe the clinical characteristics of ACL in Chinese adults and relate our experience in treating this disease.

## Methods

We retrospectively reviewed the medical records of adult patients with ACL recruited from November 1984 to August 2017 at Peking Union Medical College Hospital (PUMCH), Beijing, China. Written informed consent was obtained from each patient, and this study was approved by the Institutional Review Board of PUMCH.

Detailed demographic and clinical data were collected, including the location of ACL, symptoms, and physical examination findings. Available ultrasound, computed tomography (CT), and magnetic resonance imaging (MRI) results were reviewed. Therapeutic choices, scope of surgery, length of incision, volume of blood loss, and duration of hospital stay were recorded. Follow-up of these patients was performed in June 2019 via telephone, e-mail, or review of medical records from the last outpatient check-up.

All data were recorded and analyzed using IBM SPSS Statistics for Windows, version 22.0 (IBM Corp., Armonk, NY, USA). Descriptive data were expressed in numbers (%) for categorical variables and mean (standard deviation, SD) for continuous variables, as appropriate. Student’s t-test and Fisher’s exact test were used for continuous and categorical variables, respectively. All data tests were two-sided and a *P*-value < 0.05 was considered to be statistically significant.

## Results

A total of 12 Chinese adult patients with ACL were recruited, including seven males and five females. The demographic and clinical characteristics of these patients are shown in Table 1. The mean age at treatment was 39.1 (SD 17.3) years. The time interval from initial symptoms and first imaging evaluation to treatment were 17.8 (SD 12.2) months and 4.8 (SD 3.9) months, respectively. The mean duration of follow-up was 6.9 (SD 4.5) years until June 2019 (Table 1).

**Table 1:** Demographic and clinical characteristics of adult ACL patients

Case No.	Age	Location ^a^	Symptom ^b^	Physical sign	Evaluations ^c^	Size/cm
1	40–49	PO	Absent	Absent	CT + MRI	11.6*7.6
2	40–49	PO	Absent	Absent	US+CT + MRI	14.1*11.0
3	40–49	Left R	Absent	Absent	CT	12.0*10.0
4	20–29	HL	AP	Absent	CT	4.5*4.2
5	10–19	M	D + F	Mass via abdomen	US	20.0*19.8
6	20–29	Left R	AP	Mass via vagina	US+MRI	12.0*7.8
7	60–69	Right R	D	Mass via abdomen	US+CT	15.3*13.4
8	20–29	Right R	Absent	Absent	US+CT	15.1*6.5
9	20–29	M	Absent	Absent	US+CT	23.4*16.7
10	30–39	S	Absent	Absent	US+CT	10.1*5.1
11	70–79	Left R	AP	Absent	CT	11.1*6.7
12	40–49	Left R	AP + F	Absent	US+CT + MRI	10.8*6.0

ACL: abdominal cystic lymphangioma;

a. PO: posterior cavity of omentum; R: retroperitoneum; HL: hepatoduodenal ligament; M: mesentery; S: spleen

b. AP: abdominal pain; D: distention; F: fatigue

c. US: ultrasound; CT: Computed tomography; MRI: Magnetic Resonance Imaging

For the 12 patients with ACL, the retroperitoneal space was the most commonly affected location. Four (33%) patients had ACL affecting the left retroperitoneal space, and two (17%) had ACL in the right. Moreover, two (17%) patients were reported to have ACL in the posterior cavity of the omentum and two (17%) in the mesentery. Rare locations, namely the hepatoduodenal ligament and spleen, were each reported in two different patients respectively. None of the 12 patients reported a family history of ACL. Two patients have first-degree relatives with abdominal tumor, one with bladder tumor, the other with gastric cancer (Table 1).

Six (50%) patients were asymptomatic and had ACL incidentally detected during routine medical examination. For the other six patients who reported symptoms, abdominal pain was the leading symptom (33%). Abdominal distention, fatigue, and increased waist circumference were reported in two patients each. No patient suffered from nausea, vomiting, or acute abdomen (i.e. bowel obstruction, volvulus, and hemorrhage). Appendicitis, uterine myoma, tuberculosis, hypertension, and other conditions were observed in various patients, but no definite correlation with the ACL has been spotted. (Table 1).

Each patient underwent a detailed physical examination. Nine (75%) of the 12 did not have obvious physical signs of ACL. Only two patients had a palpable abdominal mass. One patient was initially misdiagnosed with an ovarian cyst and treated in the Department of Gynecology at PUMCH; a 10 cm × 10 cm mass was palpable under bimanual gynecological examination of this patient (Table 1).

**Fig. 1:**
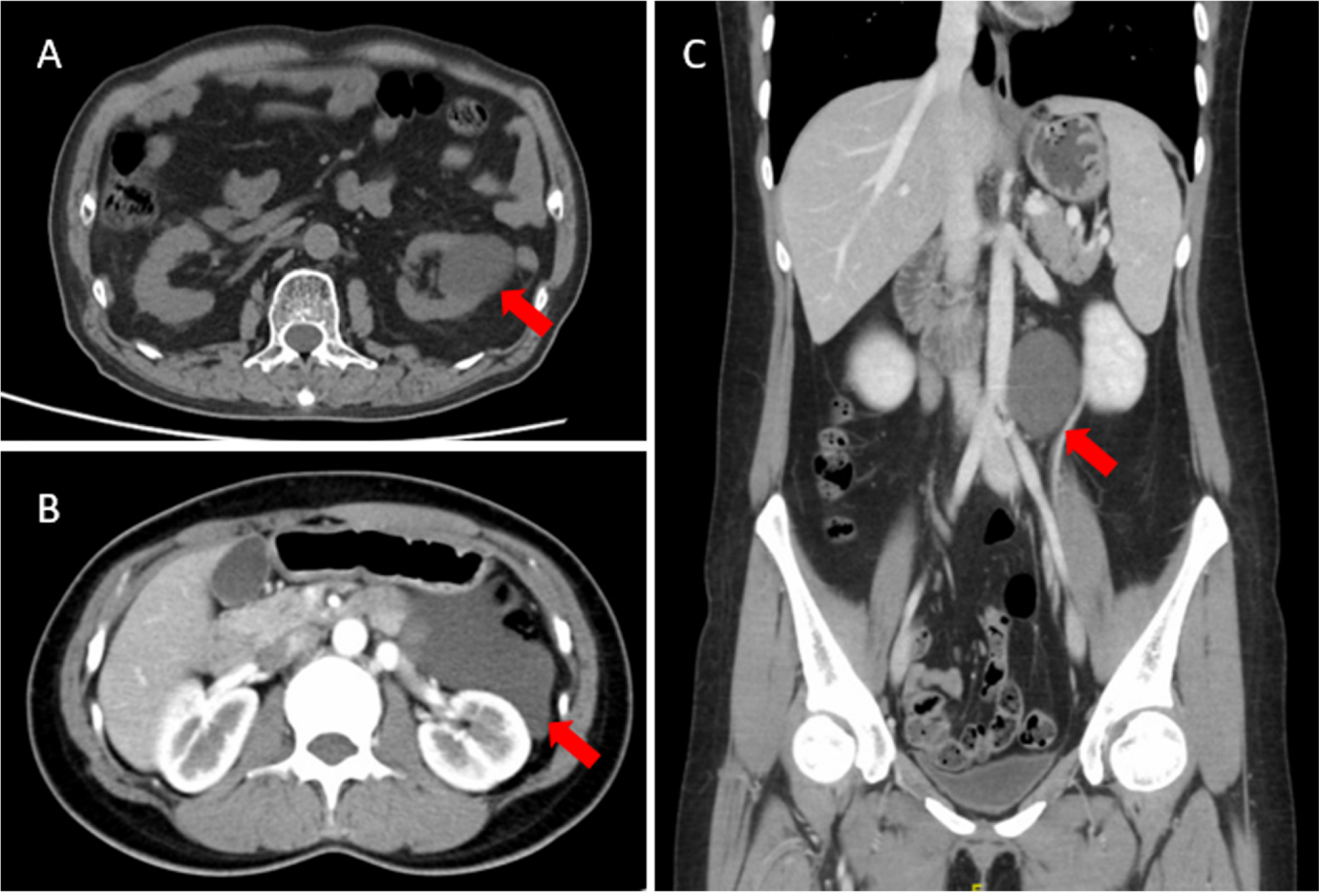
CT scans of Chinese adult patients with ACL. A. ACL close to left renal pelvis of case no. 11 (red arrow).B. ACL between spleen and stomach of case no. 3 (red arrow). C. ACL between aorta and left kidney in three-dimensional CT reconstruction of case no. 12 (red arrow). ACL, abdominal cystic lymphangioma; CT, computed tomography

**Fig. 2:**
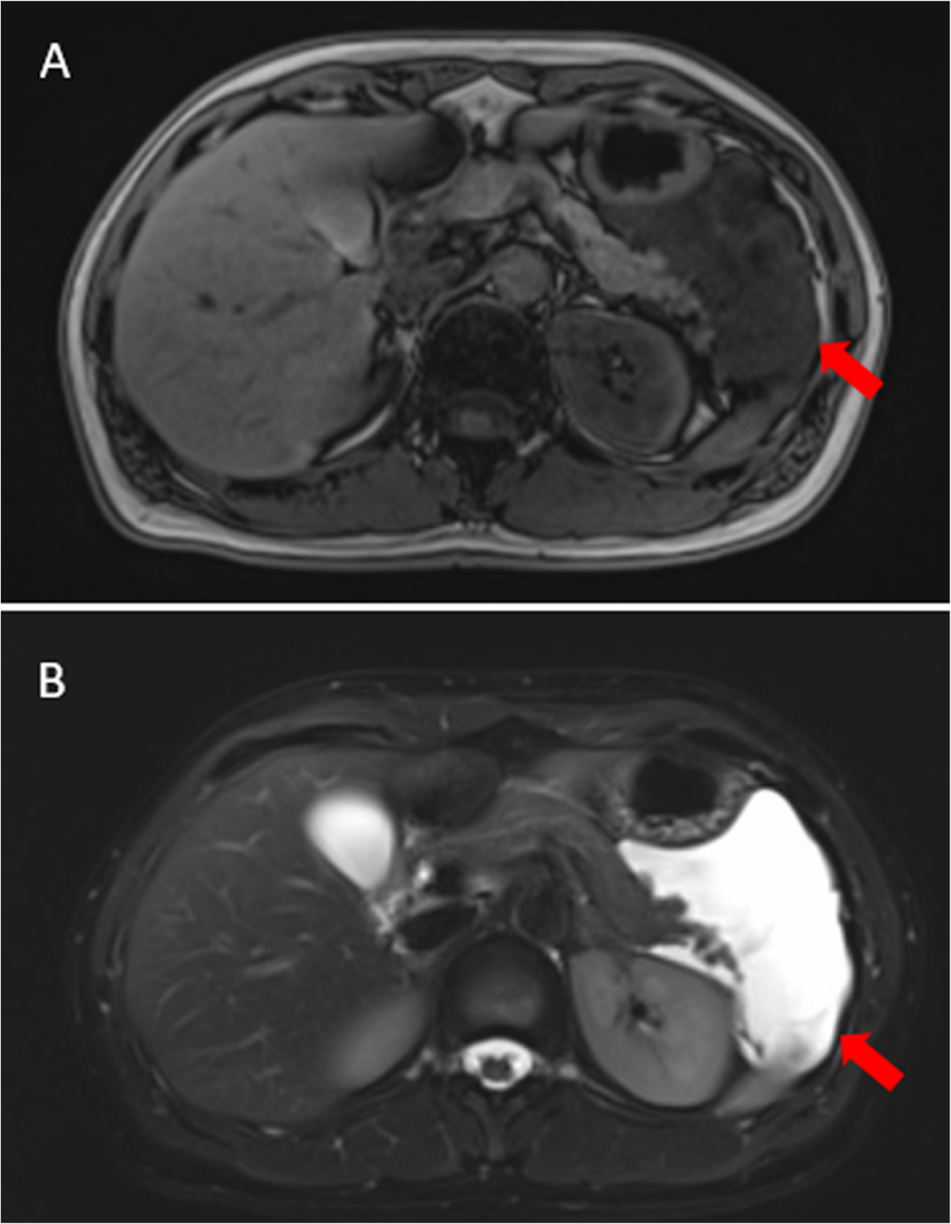
MRI of Chinese adult patients with ACL. A. Low signal in T1WI sequence of MRI of case no. 3 (red arrow). B. High signal in T2WI sequence of MRI of case no. 3 (red arrow). ACL, abdominal cystic lymphangioma; MRI, magnetic resonance imaging; T1WI, T1-weighted image; T2WI, T2-weighted image

Diagnostic imaging procedures included ultrasound (67%), CT scan (83%), and MRI (33%). Only two patients were absent from CT scan. One patient was diagnosed in the 1980s when CT was not available in our hospital. The other patient was misdiagnosed with an ovarian cyst before surgery and CT scanning was commonly not performed for these patients. The longest diameter of ACL on ultrasound, CT scan, and MRI was 12.1 (6.2) cm, 9.7 (4.3) cm, and 11.3 (3.7) cm, respectively. A paired t-test of the longest diameter was calculated for patients who underwent more than one imaging procedure. The *P*-values were 0.473, 0.440, and 0.180 for the comparison of ultrasound and CT, CT and MRI, as well as ultrasound and MRI, respectively; all were non-significant (Table 1, Figs. 1 and 2).

Ten patients underwent surgery: six (50%) underwent open surgery and four (33%) underwent laparoscopic surgery. None required urgent surgical intervention. Aspiration under CT guidance was performed in two patients, and no sclerosing agents were injected into the cyst.

Histopathologic study matched the diagnosis of CL in 10 surgical patients. Dilated lymphatic vessels lined with endothelial cells and abundant lymphoid tissues were observed in the specimens. The diagnosis was validated by immunohistochemistry, in which D2–40, a marker for lymphatic vessels, was positive.

**Fig. 3:**
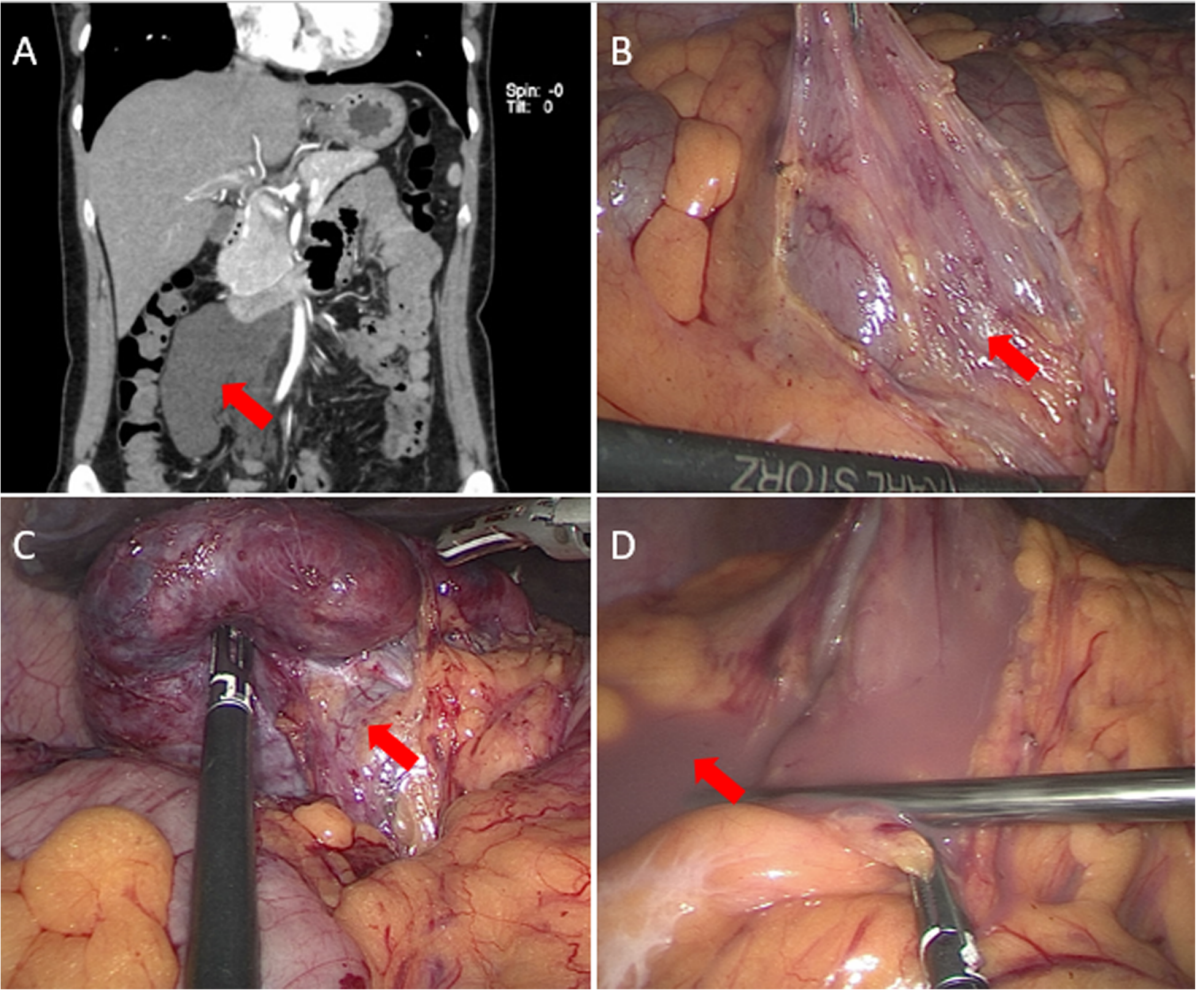
CT reconstruction and intraoperative images of a mesenteric ACL. A. ACL of mesentery in three-dimensional CT reconstruction of case no. 9 (red arrow). B. ACL in mesentery (red arrow). C. Root of mesenteric ACL (red arrow). D. Clear fluid in mesenteric ACL (red arrow). ACL, abdominal cystic lymphangioma; CT, computed tomography

Treatments and outcomes for the patients in our cohort are shown in Table 2. For two patients who underwent aspiration, a diagnosis was made by the typical imaging features and laboratory-confirmed chylous drainage fluid. For the ten surgical patients, complete excision was accomplished in eight patients and partial excision in two patients, one with ACL in the mesentery, and the other with ACL in the spleen. Surgical blood loss was less than 50 mL in the eight patients. Two patients, who had open surgery, had relatively larger volumes of blood loss: 1600 mL (mesenteric ACL) and 200 mL (splenic ACL). The former operation was performed in 1984 when the ultrasonic scalpel and other hemostatic equipment were not widely used. The latter one was because of inadvertent rupture of the mesenteric vessels in dealing with adhesions between the cyst and the mesentery. For six patients who underwent open surgery, the mean length of the incision was 16 (7.3) cm. And this length was 8.3 (6.6) cm longer than the longest diameter of the imaging procedures (ultrasound, CT, and MRI; Table 2 and Fig. 3).

**Table 2:** Treatment and outcome of adult ACL patients

Case No.	Treatment	Surgery scope	Bleeding	Hospital stay, Pre-operation/ Post-operation	Follow-up
1	Lap	Complete	200 ml	2/5	10 years
2	LS	Complete	≤50 ml	5/10	2 years
3	Lap	Complete	≤50 ml	3/7	Recurrent
4	Lap	Complete	≤50 ml	8/7	9 years
5	Lap	Partial	1600 ml	16/16	Loss
6	Lap	Complete	≤50 ml	2/6	16 years
7	Asp	NA	≤50 ml	10/5	Loss
8	Lap	Complete	≤50 ml	7/7	9 years
9	LS	Complete	≤50 ml	7/4	3 years
10	LS	Partial	≤50 ml	3/6	Relapse
11	LS	Complete	≤50 ml	2/3	8 years
12	Asp	NA	≤50 ml	7/6	10 years

ACL: abdominal cystic lymphangioma; NA: not acquired; Lap: Laparotomy; LS: Laparoscopic surgery; Asp: Aspiration under computed tomography guidance

The median hospital stay was 12.8 (SD 6.9) days, comprising 6.0 days before treatment and 6.8 days after treatment. Patients who underwent laparotomy and patients with ACL of the mesentery and retroperitoneum had relatively longer postoperative hospital stays (8.0 vs. 5.7, *P* = 0.255; 7.1 vs. 6.3, *P* = 0.583). One patient developed hypokalemia on postoperative day 1 and resolved with potassium supplementation. No other severe complications were reported. With an average 6.9 years of follow-up, no recurrence was noted among eight patients. One patient with partial cystectomy of the ACL involving the spleen reported a slowly enlarging tumor remnant. The other patient reported disease recurrence 1 year after radical excision of a retroperitoneal cyst. Two patients were lost to follow-up, one who had undergone partial resection of a mesenteric ACL, and the other who had undergone aspiration of a retroperitoneal ACL (Table 2).

## Discussion

This is the first and largest Chinese adult cohort of patients with ACL described in the literature to date. To our knowledge, there have only been two cohorts of adults with ACL published in the English literature [7, 8]. By comparing our results with other cohorts, similarities and differences between these cohorts could be drawn (Table 3).

**Table 3:** Comparison of Chinese adult ACL cohort with other published cohorts

Cohorts	Tunisian (7)	French (8)	Chinese
Patients, n	20	9	12
Male, %	40	10	58
Age at treatment, years	46	36	39
Follow-up, months	5	28	83
Locations			
Retroperitoneum, %	23	66	50
Omentum, %	14	11	17
Mesentery, %	23	22	17
Spleen, %	7	0	8
Symptoms			
Asymptomatic, %	20	22	50
Abdominal pain, %	75	88	33
Acute abdomen, %	10	0	0
Physical signs			
Absent, %	40	44	75
Mass touched on abdomen, %	60	55	17
Imaging methods			
Ultrasound, %	90	100	67
CT, %	85	77	83
MRI, %	0	22	33
Open surgery, %	65	0	50
Laparoscopic surgery, %	35	100	33
Aspiration under CT guidance, %	0	0	17
Complete excision, %	90	100	80
Partial excision, %	10	0	20
Follow-up			
No relapse, %	90	100	80
Relapse, %	10	0	20

The clinical symptoms of ACL in adults are highly polymorphic. A huge tumor may induce abdominal pain and compression symptoms. Rarely, weight loss, symptoms of Infection, hydatid cyst, intracystic hemorrhage, and other conditions have also been reported [7, 9]. In this cohort, half of the patients did not complain of any symptoms before incidental diagnosis of the cyst during an annual medical examination. This ratio is slightly but not significantly higher than other cohorts, partly due to socioeconomic development and increased awareness of health promotion (50% vs 21%, *P* = 0.128). ACL in adults may lead to misdiagnosis of masquerading diseases [10]. In this study, only two patients were diagnosed with ACL before surgery from the typical imaging features.

Among the diagnostic imaging methods, ultrasound was regarded as the first-line examination modality and suitable for disease screening. A thin, clear boundary with strong echo, hypoechoic fluid in the center, and no blood flow signal in color Doppler flow imaging are all typical features of ACL. Noticeably, when the cyst is complicated by internal bleeding, it might also have echogenic content, which was not observed in this cohort. Whether the cyst has a septum or not is not deemed to have diagnostic value. For 12 ACL patients, five cysts were multilobular under ultrasound and were verified histopathologically. The CT scan and MRI are also excellent diagnostic tools. A typical ACL under CT is usually a low-density cyst, with an average of 19.7 Hounsfield units in our Chinese cohort. The shell of the cyst is normally glossy and regularly shaped, while the homogeneous content never takes the contrast. For MRI, ACL presents as a low signal mass in the T2-weighted image sequence and a high signal in the T1-weighted image sequence in all patients who underwent the study. As with CT, no contrast enter the cyst in MRI examinations either. According to our experience, MRI was normally used as an adjunct tool to distinguish the possible origin and positional relationship of the cyst with adjacent organs. The differences in longest diameter of each imaging method are not significant, which indicates the accuracy and stability of these examinations for evaluating ACL. An accurate diagnosis of ACL may be established by a combination of these imaging modalities, but final confirmation should be done by pathologists. Other imaging investigations, such as lymphoscintigraphy, were not performed at our medical center because of its potentially poor diagnostic value [11].

The size of the ACL in this study varied from 5 × 4 cm to 23 × 17 cm, which was consistent with the range of 3–20 cm reported in the literature, except for the largest one in our cohort [7, 9]. The growth rate could be estimated in three patients from detailed records before admission to our center. An average growth of 1.6 cm annually was observed. In reviewing the medical records of the 12 adult patients with ACL, we identified no tendency for the cyst volume to decrease without intervention.

In the absence of prospective controlled studies, the indication for surgery in adult ACL patients was not standardized. Other reports recommend a watch-and-wait approach as a general rule for asymptomatic patients [7, 8]. In our center, surgery was recommended for symptomatic patients. However, because ACL may also result in acute abdomen such as internal bleeding or volvulus, and spontaneous regression of the cyst is essentially impossible, therapeutic choices for asymptomatic patients are relatively flexible [12]. When infection or malignancy is suspected, surgery should be performed. Moreover, surgery should also be considered when patients have intense anxiety and a strong preference for surgery, irrespective of the presence or absence of symptoms.

Both laparotomy and laparoscopic surgery were reported to treat ACL with acceptable outcomes [13, 14]. Based on our experience, open surgery was preferred for larger ACLs because of the complexity and uncertainty of the procedure. A longer duration of hospital stay and larger volume of blood loss may be disadvantages of laparotomy according to our data, but more studies are needed to confirm this assumption because of the relatively small cohort size. Both the retroperitoneoscopic and anterior approaches for laparoscopic surgery were performed. A retroperitoneoscopic approach was more suitable for an ACL that was close to the urinary system, as in case no. 11 (CT scan shown in Fig. 1A).

Percutaneous interventions are controversial for ACL treatment [15]. Some researchers reported that sclerotherapy was effective for symptom resolution and volumetric reduction for pediatric patients with ACL [16]. More studies argued that aspiration, with or without injection of sclerosing substances, had a higher relapse rate in ACL [17, 18]. Other than the 10 patients with ACL who underwent surgery, aspiration procedures under CT guidance were performed for two patients in this study. Because aspiration would only drain the fluid in the cyst rather than resect the lesion completely (which may result in disease relapse), surgery should still be the first choice for the treatment of ACL in adults. Aspiration was only conducted when surgical contraindications existed or for socioeconomic reasons. In this study, one patient (case no. 12) refused surgery because of the cost of the operation. No relapse was observed during 10-year follow-up. The other patient (case no. 7) had contraindications to anesthesia and thus was not a surgical candidate. Unfortunately, this patient was lost to follow-up. Therefore, aspiration and drainage may only be considered as an adjunctive therapy in the treatment plan of difficult-to-operate lymphangiomas. A specific drug, oral mammalian target of rapamycin inhibitor, sirolimus, was also reported to be an effective treatment for extensive lymphatic malformation, but this drug was not used for Chinese ACL patients [19].

Radical resection was considered to have a lower recurrence rate than partial excision [20, 21]. For eight patients with complete resection of ACL, only one patient had disease recurred after 6.9 years of follow-up (range 2–16 years). The follow-up duration is longer than other cohorts of adult patients with ACL [7, 8]. Complete resection should be attempted initially if there is no obvious contraindication for surgical intervention. Because ACL is usually a benign tumor, resection of other normal organs, such as the small intestine, should be avoided. For all 12 patients in our Chinese cohort, associated bowel resection was only performed in one patient. However, splenectomy is increasing in popularity for the treatment ACL affecting the spleen [22]. Splenic ACL is a slow-growing neoplasm, usually seen during childhood and rarely observed beyond 20 years of age [23]. In this study, only one patient had splenic ACL, perhaps because of the relatively small cohort size. That patient only underwent a partial resection, and it was reported that the diameter of the remaining ACL had grown 3 cm during the previous 3 years. Therefore, this case also supported the contention that splenectomy might be the first choice for splenic ACL.

Our study had some evident limitations. First, it was a retrospective study, even though we used different methods to gather information, conditions of recall bias and missing data still existed. Second, this cohort only recruited 12 adult ACL patients. Further studies with more patients enrolled are still needed. Third, our hospital is a national comprehensive medical center in China, cases presented in this study may not be completely representative for all Chinese adult ACL patients.

## Conclusions

In summary, we report the first cohort of Chinese adults with ACL, to the best of our knowledge. We have provided detailed demographic and clinical data for these patients and compared the differences between our Chinese cohort and other cohorts. With the longest duration of follow-up, our findings support the conclusion that complete surgical excision of the cyst is recommended. We also provided evidence that ACL in adults could be detected by typical imaging features and treated in a timely manner. This cohort enriches the knowledge of the disease spectrum for ACL and deepens our understanding of the disease.

## Data Availability

The datasets used and analysed during the current study are available from the corresponding author on reasonable request.

## Abbreviations

CL: Cystic lymaphangioma
ACL: Abdominal cystic lymphangioma
PUMCH: Peking Union Medical College Hospital
CT: Computed tomography
MRI: Magnetic Resonance Imaging
ml: Milliliter
cm: Centimeter
